# Genotype x environment interaction and yield stability of soybean (Glycine max l.) genotypes in multi-environment trials (METs) in Nigeria

**DOI:** 10.1016/j.heliyon.2024.e38097

**Published:** 2024-09-18

**Authors:** Abush T. Abebe, Adeyinka S. Adewumi, Moses Adeolu Adebayo, Aondover Shaahu, Hapson Mushoriwa, Tunrayo Alabi, John Derera, Afolabi Agbona, Godfree Chigeza

**Affiliations:** a International Institute of Tropical Agriculture, Ibadan, Nigeria; b Department of Crop and Animal Science, Ajayi Crowther University, Oyo Town, Nigeria; c National Soybean Program, National Cereals Research Institute (NCRI), Badeggi, Nigeria; d Texas A&M AgriLife Research Center, Weslaco, TX, USA; e International Institute of Tropical Agriculture, Southern Africa Research and Administration Hub (SARAH) Campus, Lusaka, Zambia

**Keywords:** Genotype x environment interaction, GGE biplot, AMMI, WAASB index, ASV index multi-locational trials, Selection

## Abstract

Genotype × environment interaction (GEI) poses a critical challenge to plant breeders by complicating the identification of stable variety (ies) for performance across diverse environments. GGE biplot and AMMI analyses have been identified as the most effective and appropriate statistical techniques for identifying stable and high-performing genotypes across diverse environments. The objective of this study was to identify widely adapted and high-yielding soybean genotypes from Multi-Locational Trials (MLTs) using GGE and AMMI biplot analyses. Fifteen IITA-bred elite soybean lines and three standard checks were evaluated for stability of performance in a 3 × 6 alpha lattice design with three replications across seven locations in Nigeria. Significant (p < 0.001) differences were detected among genotypes, environments, and GEI for grain yield, which ranged between 979.8 kg ha^−1^ and 3645 kg ha^−1^ with a mean of 2324 kg ha^−1^. To assess the stability of genotypes, analyses were conducted using the general linear method, GGE, and the Additive Main Effect and Multiplicative Interaction (AMMI) approach, as well as WAAS and ASV rank indices. In the GGE biplot model, the first two principal components accounted for 67.4 % of the total variation, while in the AMMI model, the first two Interaction Principal Component Axes (IPCA1 and IPCA2) explained 73.20 % and 11.40 % of the variation attributed to genotype by environment interaction, respectively. GGE biplot identified G10 and G16 as the most stable and productive genotypes, while WAASB index revealed G16, G10, G9, G4 and G2 as the most adaptive, stable and productive genotypes across locations, and ASV identified G9, G13, G4, G14 and G10 as the most stable and productive.

Consequently, genotypes G2, G4, G9, G10 and G16 displayed outstanding and stable grain yield performance across the test locations and are, therefore, recommended for release as new soybean varieties suitable for cultivation in the respective mega environment where they performed best. More importantly, the two genotypes are recommended for recycling as sources of high-yield and yield stability genes, and as parental lines for high-yield and stable performance for future breeding and genomic selection.

## 1 Introduction

As a crop with multiple importance, soybean has untapped potential in supporting economic growth in Africa, serving as an affordable source of high-quality protein (40 % of the grain) and vegetable oil (21 %). Foyer et al. [1] commended soybean as an ideal crop to fill in the widening gap of the need for protein, edible oil and animal feed in Africa. Plant protein-based diets can replace meat-based diets without compromising nutrition because of well-balanced essential amino acids except sulphur-containing amino acids. Increased demand for soy cake, a nutritionally rich feed supplement for the poultry and livestock industries, was among the major factors that caused increased demand for soybean that also increased the market opportunity for the soybean grain from small-holder farmers, as they sell it to traders and processors. In the context of smallholder farming, where farm inputs and other resources are limited, soybean provides multiple benefits in the crop production systems by fixing atmospheric nitrogen and making it available for crops’ use [2]. Soybeans also plays an important role in the diversification of crops in the agro-ecosystems through rotation with cereals, which is important in breaking the pest and disease cycles [3,4]. Leguminous crops, such as soybean, are ideal for rotation with cereals in areas where continuous cereal sole cropping is a common practice.

In recent years, soybean demand has witnessed a remarkable increase in Nigeria. This is stimulated by the growing demand from both the local and international markets. The Nigerian soybean crush for oil capacity was 875,000 MT in 2023 [5], while the production of soy meal has grown from 177,000 MT in 2011 [6] to 680,000 MT in 2023 [5]. The total Nigerian soybean demand was estimated to be 2.2 million tons in 2016, which was far above the total production of 680,000 MT in the same year [6], and the demand has been projected to grow to 3.0–3.5 million MT by 2024 [7].

Despite the growing demand in the country, soybean production has been growing at a slow rate over the years. The volume of production that was estimated as 580,000 MT in 2007 has increased to only 980,000 MT in 2021, which was an average annual growth of 28,571.43 MT for 14 years [8]. The same report indicated that soybean productivity increased from 0.91 t ha^−1^ in 2007 to 0.93 t ha^−1^ in 2021 by a slim margin of 2.2 % only, indicating that the productivity remained nearly unchanged over the same period. This also shows the increase in production has been solely due to increased production area, not increased yields. The average national productivity of (0.93 t ha^−1^) is very low relative to the average Africa's productivity of 1.44 t ha^−1^, the average global productivity 2.87 t ha^−1^ and the average productivity of the best soybean-producing countries such as USA (3.45 t ha^−1^) and Brazil (3.44 t ha^−1^), in 2017 [8]. Interestingly, yields beyond 3.0 t ha^−1^ have been achieved in the research plots of the IITA soybean program, IITA GoSeed demonstrations, and on the National Research Institute (NCRI) plots, suggesting that there is high potential for improving soybean yields in Nigeria through increased adoption of high yielding varieties that respond to the best agronomic practices. Limited availability of improved varieties for production, the slow rate of replacement of old varieties with new and high-yielding and resilient varieties, and the slow rate of adoption of new, improved varieties have been identified as some of the major factors that significantly contributed to the low productivity of the crop in Nigeria. The contributions of environmental factors, such as moisture stress, low soil fertility, high temperature, and erratic rain distribution during the growing period also account for the low soybean yields in Nigeria. The development and evaluation of new soybean genotypes across wider and diverse environmental conditions is, therefore, essential for identifying soybean varieties with stable and superior yield performance, which are also resilient to adverse environmental conditions. On another note, the multi-location trial results can show the varieties that stand out in specific environments. As such, these can be recommended for specific adaptation.

Breeders often use performance for yield and its associated traits across different environments to select crop cultivars with superior performance and stability. The focus of crop improvement approaches should place emphasis on reducing risks, stabilizing yield, reducing costs, and maximizing income [9]. Factors such as pathogenic infections, humidity, soil texture and fertility, precipitation, and temperature can all cause yield fluctuations due to the reaction of genotypes to these variable environmental factors [10]. These environmental fluctuations, which are summed up as genotype × environment interaction (GEI), have been observed to significantly influence productivity in various crops [11,12] they negatively affect heritability [13] due to high GEI, thereby complicating selection for crop improvement [14]. Assessing the adaptability and stability of genotypes is crucial in plant breeding. Yan and Kang [13], Khush [15], and Duvick [16] emphasized the important implications of any form of tolerance to biotic and abiotic stresses for stable performance of genotypes across diverse environmental conditions. Therefore, it is important to evaluate the performance of soybean genotypes in multi-environment trials (METs) to assess the influence of GEI and identify superior and stable genotypes across locations. Analyzing METs allows breeders to identify and comprehend the impact of GEI on the final performance and ranking of a genotype. Several methods have been utilized for the assessment of the adaptability and stability of genotypes across various environments. These methods include the joint regression method introduced by Finlay and Wilkinson [17], stability models developed by Eberhart and Russell [18], the additive main effects and multiplicative interaction (AMMI) method proposed by Gauch [19], and the genotype main effects and genotype by environment interaction (GGE) biplot method developed by Yan et al. [20]. AMMI and GGE have been considered the best techniques for interpreting GEI data and are more commonly utilized than other stability procedures, as noted by Bhartiya et al. [21] and Khan et al. [22]. The AMMI method integrates ANOVA and principal component analysis to generate a biplot that illustrates the connection between genotype means and the first principal component, as highlighted in research by Mitrović et al. [23] and Bocianowski et al. [24]. The GGE biplot technique was found to be the most preferred and widely used multivariate method in METs of various agricultural crops [20,25,26], including soybean [14,27]. GGE biplot analysis was used in several studies to identify the most suitable environment, rank genotypes, and determine discriminative and representative environments [10] based on a biplot technique employing graphical analysis of MET [14]. Compared to other methods, GGE biplot was found to be more effective in identifying stable and superior cultivars across multiple environments [28].

Therefore, the current study was designed to analyze multi-environments data using GGE biplot approach to identify superior and stable soybean genotypes across seven diverse locations in Nigeria that can be recommended for release and commercial production. Specifically, this study sought [1] to determine the most discriminating and representative testing environments [2], to determine the best stable and productive genotypes across the test environments and [3] to determine the adaptive genotype in a particular location.

## 2 Materials and methods

### 2.1 Plant materials and study locations

**Fig. 1:**
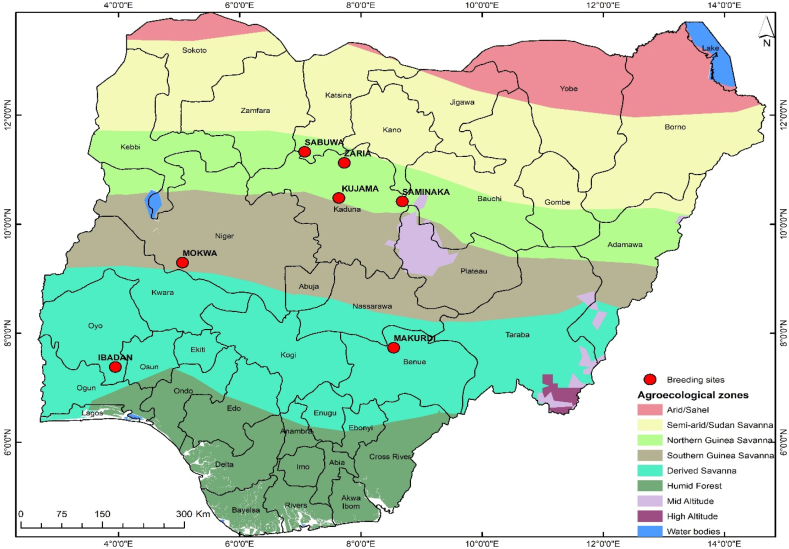
The locations used in the multi-location yield trials and the different agro-ecological zones the locations representing.

Fifteen [15] elite soybean lines developed by the IITA soybean breeding program and three standard checks (Table 1) were evaluated in seven diverse locations (Ibadan, Zaria, Kujama, Makurdi, Mokwa, Sabuwa and Saminaka) in Nigeria in the 2019 rainy season. Three medium maturing (that matures between 100 and 120 days) check varieties were used in the study, of which the two checks i.e., TGx 1951-3F, and TGx 1989–19F were released in 2014, whereas TGx 1448-2E was released in 1992 for high yield and disease resistance in Nigeria. The varieties TGx 1989–19F and TGx 1951-3F were reported to be resistant to Asian soybean rust (ASR) and Cercospora leaf spot (https://www.seedportal.org.ng/variety.php?cropid=4&task=view) and TGx 1989–19F reported for possessing ASR resistant genes (Rpp1 and Rpp3) [29]. The agro-ecological descriptions of the seven locations are provided in Table 2 and Fig. 1. In addition, the soil description of the test locations is presented in Table 3.

**Table 1:** Pedigree information Fifteen [15] elite soybean lines used in the study.

Gen	Pedigree
G16	TGx2110-09FN
G2	TGx2103-04FN
G12	TGx2077 -5FN
G10	TGx2108-01FN
G3	TGx2110-08FN
G9	TGx2103-01FN
G18	TGx2065 -5FZ
G4	TGx2111-04FN
G14	TGx2103-02FN
G13	TGx2111-05FN
G17	TGx2110-10FN
G11	TGx2108-02FN
G6	TGx2103-05FN
G7	TGx2111-10FN
G15	TGx2103-03FN
**Checks**	
G1	TGx1951-3F
G8	TGx1989-19F
G5	TGX-1448-2E

**Table 2:** Agro-ecological descriptions of the test locations used in the study.

	Sites	Latitude	Longitude	Mean Ann. RF (mm)	Mean max. Temp.	Mean min. Temp.	Alt (m asl)
1	Ibadan	7.37750	3.94700	1159.0	31.8	22.1	227.0
2	Zaria	11.12470	7.72540	951.8	31.9	18.6	640.0
3	Kujama	10.48020	7.63580	1262.8	31.3	18.7	683.0
4	Makurdi	7.73220	8.53910	1244.2	32.8	22.6	93.0
5	Mokwa	9.29280	5.05470	1033.8	33.2	21.5	155.0
6	Sabuwa	11.32670	7.07140	1009.0	31.7	18.2	677.0
7	Saminaka	10.41658	8.68200	1299.6	30.8	17.7	769.0

**Table 3:** Soil characteristics of the test locations used in the study.

	Extractable potassium (mg kg^−1^)	Organic Carbon (g kg^−1^)	Phosphorus content	pH (H2O)	Total organic N (g kg^−1^)	Soil texture
Ibadan	142	8.6	11.6	6.18	72	Sandy loam
Zaria	65	5.5	8.1	6.29	44	Silt loam
Kujama	67	5.4	5.8	5.90	51	Sandy loam
Makurdi	62	5.4	8.2	6.19	79	Sandy loam
Mokwa	43	4.8	8.5	6.13	64	Sandy loam
Sabuwa	59	6.2	6.0	5.87	46	Sandy loam
Saminaka	93	5.6	8.5	5.80	51	Loam

### 2.2 Experimental design and management

The experiment was laid out in 3 × 6 alpha lattice design, replicated three times at each location. Plot size comprised four rows, each 4m long, spaced at 50 cm between rows and a spacing of 5 cm between plants within a row. Two seeds were planted per hill, and later thinned to one plant per hill two to three weeks after sowing. The NPS and Urea fertilizers were applied at the recommended rates of 122 and 50 kg ha^−1^ during sowing and flowering, respectively. Manual weeding was done three times in the growing season to manage weeds, and all the remaining agronomic practices were applied as per the recommendation for soybean.

### 2.3 Data collection

Morphological characterization was carried out on traits such as (fodder yield, nodulation score (1–5 scale), shattering score (1–5 scale), pod length (cm), plant height (cm), lodging score (1–5 scale), days to 95 % maturing (number of days from planting to 95 % pod maturity), days to flowering (number of days from planting until at least one flower on the 50 % of the plants flower), 100 seeds weight (gm) at different growth stages from 10 randomly selected and tagged plants from each plot and each replication according to the soybean descriptor of IBPGR [30].

### 2.4 Statistical analyses

Separate analysis of variance (ANOVA) was conducted on the data collected on all the morphological traits on plot basis for each location. The homogeneity of error variances of the individual locations was tested and found non-significant, indicating the possibility to proceed with combined ANOVA across locations using lme4 package in R [31]. Locations and replications within location were considered random effects, whereas the genotypes were considered as fixed effects and used to determine the significance level of genotypes (G), environments (E) and their interaction (GEI). The combined ANOVA model used in this study is provided in the following equation [1]:

(1) $$Y_{ijkl}=\mu +G_{i}+E_{j}+R_{k\left(j\right)}+B_{l\left(jk\right)}+{\text{GE}}_{ij}+e_{ijlk}$$

where Y_*ijkl*_ is the response of the ith genotype in jth environment and kth replication within the jth environment and lth block within replication; μ is the grand mean, G_*i*_ is the effect of ith genotype; E_*j*_ is the effect of jth environment; R_*k(j)*_ is the effect of kth replication within the jth environment; B_*l(jk)*_ is the effect of lth block in the jth environment and kth replication; GE_ij_ is the interaction effect of ith genotype and jth environment; and e_*ijkl*_ is the random error effect. To explore the G × E interaction effect, the multivariate stability analysis was performed graphically based on the GGE biplot model [32] using R package GGEBiplotGUI. Singular value decomposition (SVD) of the first two principal components was used to fit the GGE biplot model according to the equation [2] below:

(2) $$\text{GGE}=\sum_{K=1}^{n}\lambda_{k}\gamma_{ik}\alpha_{jk+}\rho_{ij}$$

where GGE is the matrix of the effects of genotypes added to the effects of the interaction; λ_k_ is the k-th singular value of the original matrix interactions (GE); γ_ik_ is the element corresponding to the i-th genotype in the k-th singular vector of the GE matrix column; α_jk_ is the element corresponding to the j-th environment in the k-th singular vector of the GE matrix row; and ρ_ij_ is the residual related to the adjustment. GGE biplots are graphical tools to demonstrate the G × E interaction and the classification of genotypes based on the mean and stability obtained for a trait of interest. The generated graph is based on METs evaluation for environmental stratification (who-won-where pattern), genotype evaluation (mean versus stability) and tested environment ranking (discriminatory versus representative). The classification of genotypes was established in ascending order of each stability parameter. The biplots were based on partitioning with singular value = 2, transformed (transform = 0), centered on the environment (centering = 2) and standardized with standard deviation (scaling = 0).

AMMI analysis was carried out according to Ref. [33]. Generally, the mean response of individual genotypes averaged over *b* replications within each environment is computed and used to fill a *g* × *e* matrix. Briefly, the estimate of *y*_*ij*_ is given in two steps according to the following equation [3]:

(3) $$y_{ij}=\mu +\alpha_{i}+\tau_{j}+\sum_{k=1}^{p}\lambda_{k\;}a_{ik}t_{jk\;}+\rho_{ij}+\varepsilon_{ij}$$

where λ_k_ is the singular value for the *k*-th interaction principal component axis (IPCA); a_ik_ is the *i*-th element of the *k*-th eigenvector; t_jk_ is the *j*th element of the *k*th eigenvector. A residual ρ_ij_ remains, if not all *p* IPCA are used, where p ≤ min (g−1; e−1).

The WAAS index function computes the Weighted Average of Absolute Scores [34] considering (i) all principal component axes that were significant (p < 0.05 by default); or (ii) declaring a specific number of axes to be used, according to the following equation [4]:

(4) $${WAAS}_{i}=\sum_{k=1}^{p}│{IPCA}_{ik}*{EP}_{k}│/\sum_{k=1}^{p}{EP}_{k}$$

where WAAS_i_ is the weighted average of absolute scores of the *i*th genotype; PCA_ik_ is the score of the *i*th genotype in the *k*th IPCA; and EP_k_ is the explained variance of the *k*th IPCA for k = 1,2,‥,p, considering *p* the number of significant PCAs, or a declared number of PCAs.

The WAASY index [35] is used for genotype ranking considering both the stability (WAAS) and mean performance based on the following equation [5]:

(5) $${WAASY}_{i}=\frac{\left({rG}_{i\;}*\;\theta_{Y}\right)+\left({rW}_{i\;}*\;\theta_{s\;}\right)}{\theta_{Y}+\theta_{s}}$$

where WAASY_i_ is the superiority index for the *i*-th genotype that weights between performance and stability; rG_i_ and rW_i_ are the rescaled values (0–100) for GY and WAASB, respectively; θ_Y_ and θ_S_ are the weights for GY and WAASB, respectively.

AMMI's stability value (ASV) was calculated to rank genotypes in terms of stability using the following equation [6]:

(6) $$AMMI\;stability\;value\left(ASV\right)=\sqrt{\left[\frac{SSIPCA1\left(IPCA1score\right)}{SSIPCA2}\right]}+{\left[IPCA2score\right]}^{2}$$

## 3 Results

### 3.1 Analyses of variance and performance of the genotypes

Single-site ANOVA revealed highly significant (P < 0.01) differences among the 18 soybean genotypes for grain yield in each of the test locations, except in Mokwa and Sabuwa (Table 4). Five locations (Ibadan, Makurdi, Kujama, Saminaka and Zaria), where the genotypes expressed significant differences with high heritability values that exceeded 50 % for grain yield were included in the combined analyses of variances. Bartlett's test of the homogeneity of error variances of the selected individual locations revealed non-significant differences justifying the possibility of proceeding with combined analyses of variances. Results of the combined ANOVA revealed that mean squares for grain yield were significant among genotypes (p ≤ 0.05), environments (p ≤ 0.001), and genotype × environment interaction (p ≤ 0.05) (Table 6). Grain yield of the 15 soybean genotypes and three checks varied from 1800.6 to 2747.6 kg ha^−1^ with a trial mean of 2324.0 kg ha^−1^. The topmost genotype, G16, produced grain yield of 2747.6 kg ha^−1^, which was not significantly higher than either of the top two checks, G1 (2652.2 kg ha^−1^) and G8 (2268.9 kg ha^−1^) (Table 3). The top eight genotypes significantly out-yielded the third check, G5, which produced a grain yield of 1998.1 kg ha^−1^. Although no definite pattern could be identified for the performance of the genotypes, both genotype and genotype × environment interaction effects were significant for most of the measured agronomic traits (Table 5). The fodder yield was highest (2022 kg ha^−1^), for the check variety, G5, followed by the genotypes G4 (1851 kg ha^−1^), G3 (1797 kg ha^−1^), the check varieties, G1 (1792 kg ha^−1^) and G2 (1771 kg ha^−1^). The check variety G1 produced the highest nodule score of 3.3, followed by G18 (3.1) and G7 (3.0). The highest-yielding genotype, G16, had a nodule score of 2.8, while the check varieties, G5 and G8, gave nearly the same nodule scores of 2.9 and 2.7, respectively. The second highest yielding check variety, G18 had a score of 3.0 as the most shattering susceptible genotype, followed by G3 (2.8), and the check variety, G8 (2.6). The other check varieties, G1 (1.6), and G5 (1.7), showed high shattering tolerance. The highest-yielding genotype, G16, had a shattering score of 2.1, while G14 with 1.2 was the most shattering-tolerant variety. The genotypes that showed the highest lodging susceptibility were G4 (2.1), G13 (2.0), G16 (1.9), G2 (1.9) and G1 (1.9). The best variety with good tolerance to lodging was G12 with the lowest lodging score of 1.2. The other check varieties, G5 (1.6) and G8 (1.5), expressed good levels of lodging tolerance. The latest days to flowering was 50 days for the genotypes G4, G6, G7, G11, G12, G13, G16, G17, and G18. The earliest days to flowering was 46 days for the genotypes G2 and G8. The other two checks, G1 and G5, flowered in 47 and 48 days, respectively. The genotypes G13, G4, G16, G7, G6 and G1 showed the highest 100 seeds weight of 15.8, 15.6, 15.5, 15.3 and 15.0 g respectively. The other two check varieties, G8 and G5, showed 100-seed weights of 14.8 and 13.5 g, respectively.

**Table 4:** Mean grain yield (kg ha^−1^) performance and statistic of 15 soybean genotypes and three standard checks in seven individual and across five sites in Nigeria in 2019.

Gen	Pedigree	aCombined (n = 5)	Ibadan	Makurdi	Mokwa	Kujama	Sabuwa	Saminaka	Zaria
G16	TGx2110-09FN	**2747.6**	3566.9	2952.3	1940.2	2590.8	1392.0	1971.7	3497.7
G2	TGx2103-04FN	**2539.5**	2776.7	2207.3	1998.1	2826.1	1248.4	2405.6	2827.1
G12	TGx2077 -5FN	**2460.1**	3293.9	2413.1	2057.7	2560.5	1362.7	1568.7	2683.6
G10	TGx2108-01FN	**2426.6**	2959.0	1299.1	2060.4	2859.4	1398.7	1710.3	3451.3
G3	TGx2110-08FN	**2397.8**	2779.6	2044.1	2109.3	2932.4	1338.0	2013.9	2214.3
G9	TGx2103-01FN	**2384.3**	2968.6	1753.2	1983.8	2346.5	1491.0	1640.1	3396.7
G18	TGx2065 -5FZ	**2347.4**	2448.7	3023.2	2026.6	1978.4	1486.3	1445.9	3182.5
G4	TGx2111-04FN	**2341.2**	3093.5	994.0	2039.0	2583.7	1365.1	2049.1	2874.3
G14	TGx2103-02FN	**2283.0**	3054.9	1611.3	2005.2	2388.3	1245.9	1724.0	2476.5
G13	TGx2111-05FN	**2276.3**	2952.7	979.8	2029.2	2540.5	1372.1	1925.7	2768.6
G17	TGx2110-10FN	**2264.2**	2321.5	2178.9	2033.7	2350.7	1354.8	2301.6	2038.6
G11	TGx2108-02FN	**2259.9**	3127.5	1973.2	2003.4	2536.5	1388.0	1689.2	1667.8
G6	TGx2103-05FN	**2201.5**	2595.7	930.2	2024.8	2553.6	1265.1	1777.3	2861.2
G7	TGx2111-10FN	**2183.1**	2254.0	1810.0	2074.6	2203.7	1267.3	1926.5	2525.0
G15	TGx2103-03FN	**1800.6**	1698.8	1639.7	2052.4	1610.6	1215.2	1701.4	1474.1
**Checks**									
G1	TGx1951-3F	**2652.2**	2994.2	2952.3	2074.6	2699.6	1390.7	1848.9	3499.0
G8	TGx1989-19F	**2268.9**	2471.9	1831.3	2030.1	2251.0	1248.4	1259.1	3645.0
G5	TGX-1448-2E	**1998.1**	1953.7	1015.3	2081.7	2599.3	1242.8	1487.9	2299.7
**Statistic**									
	H^2^	**0.56**	0.68	0.85	0.11	0.67	0.33	0.73	0.89
	Mean	**2324.0**	2739.5	1867.1	2034.7	2467.3	1337.4	1802.6	2743.5
	LSD_0.05_	**554.5**	938.1	816.2	333.7	645.2	341.2	505.7	650.2
	CV (%)	**21.8**	25.0	28.5	30.2	19.2	26.7	19.8	15.1
	Gen	**∗**	∗∗	∗∗∗	ns	∗∗	Ns	∗∗	∗∗∗
	G x E	**∗∗∗**	–	–	–	–	–	–	–
	Env	**∗**	–	–	–	–	–	–	–

LSD = Least significant difference; CV = Coefficient of variation; Gen = Genotype; G x E = Genotype by Environment Interaction; Env = Environment; ∗,∗∗,∗∗∗ Mean squares significant at p ≤ 0.05, 0.01. and 0.0001, respectively; ns = mean squares not significant.

a Mokwa and Sabuwa were excluded from the combined ANOVA.

**Table 5:** Trait means of the 15 IITA-bred soybean genotypes and three checks evaluated across seven test environments in Nigeria in 2019.

Gen	FODY	NOD	SHAT	PODLT (cm)	PLHT (cm)	LODG	DMAT (days)	DFF (days)	100WT (g)
**G1**	1792	3.3	1.6	11.5	55.8	1.9	103	47	15.0
**G2**	1771	2.8	1.5	11.5	50.2	1.9	104	46	14.8
**G3**	1797	2.4	2.8	12.3	53.6	1.6	103	49	14.0
**G4**	1851	2.1	2.1	13.2	58.3	2.1	104	50	15.6
**G5**	2022	2.9	1.7	10.7	50.2	1.6	105	48	13.5
**G6**	1289	2.5	1.7	10.7	50.6	1.5	105	50	15.3
**G7**	1460	3.0	1.8	13.4	56.7	1.7	105	50	15.5
**G8**	1621	2.7	2.6	11.9	48.6	1.5	101	46	14.8
**G9**	1244	2.7	1.7	10.5	49.4	1.5	104	49	13.8
**G10**	1469	2.9	1.5	11.1	51.6	1.6	104	48	13.2
**G11**	1229	2.4	1.9	12.0	50.5	1.6	104	50	14.2
**G12**	1280	2.6	1.7	13.1	49.5	1.2	103	50	12.6
**G13**	1271	2.6	1.9	13.5	58.4	2.0	103	50	15.8
**G14**	1222	2.7	1.2	10.7	49.3	1.5	104	48	12.7
**G15**	1336	2.5	1.7	10.7	47.5	1.5	103	48	13.6
**G16**	1275	2.8	2.1	12.0	49.1	1.9	103	50	15.5
**G17**	1094	2.4	1.9	10.0	46.4	1.5	106	50	15.5
**G18**	982	3.1	3.0	13.4	49.5	1.4	98	50	14.8
**Mean**	1444.7	2.7	1.9	11.8	51.4	1.6	104.5	48.8	14.5
**SE**	92.2	0.2	0.2	0.5	1.5	0.1	5.5	0.4	0.3
**CV (%)**	31.3	26.2	41.5	20.2	17.0	31.4	32.4	4.8	7.8
**Gen**	∗∗∗	∗	∗∗∗	∗∗∗	∗∗∗	∗∗	ns	∗∗∗	∗∗∗
**G x E**	∗∗∗	Ns	∗∗∗	∗∗∗	∗∗∗	∗∗∗	ns	∗∗∗	∗∗∗

Gen = Genotype, Fody = Fodder yield, Nod = Nodulation score (1–5 scale), Shat = Shattering score (1–5 scale), PODL = Pod length (cm), PLHT = Plant height (cm), LODG = lodging score, DMAT = days to 95 % maturity, DFF = Days to flowering, 100WT = 100 seeds weight (gm), SE = Standard error; LSD = Least significant difference; CV = Coefficient of variation; Gen = Genotype; G x E = Genotype by Environment Interaction; Env = Environment; ∗,∗∗,∗∗∗Mean squares significant at p ≤ 0.05, 0.01. and 0.0001, respectively; ns = mean squares not significant.

**Table 6:** Sums of squares, mean squares, and percentages of variation explained by different sources of variation for grain yield (kg ha^−1^) measured in 15 soybean genotypes and three checks across seven environments during 2019 rainy season in Nigeria.

Source of Variation	DF	SS	MS	Prob	% Variation
BLK(ENV)	14	20.7836	1.484543	.^a^	
GEN	17	28.1363	1.655077	.^a^	9.8
ENV	6	90.41814	15.06969	.^a^	31.6
GxE	102	85.96563	0.8428	.^a^	30.1
Error	238	60.68	0.255		21.2
TOTAL	377	285.984			

DF = Degree of Freedom; SS = Sum of Squares; MS = Mean of Squares; BLK = Block; GEN = Genotypes; ENV = Environment; GxE = Genotype-Environment Interaction.

a Mean squares significant at p ≤ 0.001.

Partitioning the total sums of squares for grain yield revealed that genotypes accounted for less than 10 % of the variation (Table 5). Environment and GEI each accounted for 30 % of the total variation. These results necessitate further investigation into the influence of environments on the performance of the soybean genotypes using genotype and genotype × environment interaction (GGE) analyses.

### 3.2 Representativeness and discriminating ability of the test environments

**Fig. 2:**
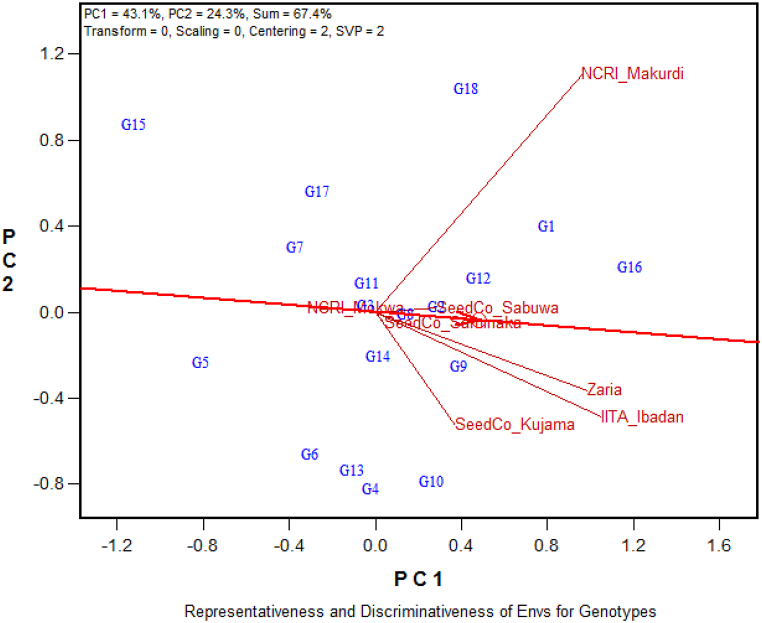
GGE biplot showing the discriminating ability and representativeness of the seven test environments PC = Principal Component.

A biplot showing the representativeness and discriminating ability of each of the seven test environments is presented in Fig. 2. Considering the length of the environment vectors, which is a measure of the discriminating ability of the environments, the most discriminating or informative environment was Makurdi, followed by Ibadan, Zaria, and then Kujama. The remaining three environments namely, Mokwa, Sabuwa and Saminaka, were non-discriminating and, therefore, offered little information about the performance of the soybean genotypes for grain yield in this study (Fig. 2). The average-environment-axis (AEA) is the pointer to the average environment represented by the small circle at the end of the arrow pointing in the positive direction of the horizontal line that passes through the origin of the biplot (Fig. 2). Among the four environments (Makurdi, Ibadan, Zaria, and Kujama) that were informative, Zaria was the most representative because of its smallest angle with AEA, whereas Makurdi, though being the most discriminating environment, was also the least representative because of its largest acute angle with AEA (Fig. 2). Zaria and Ibadan were both discriminating and representative considering the length of their vectors and acuteness of their angles with AEA, respectively, and suitable for selecting broadly adapted soybean genotypes in this study. On the other hand, the most discriminating Makurdi environment will be ideal for selecting specifically adapted genotypes due to its discriminating ability.

### 3.3 Mean yield and stability of performance of the soybean genotypes

**Fig. 3:**
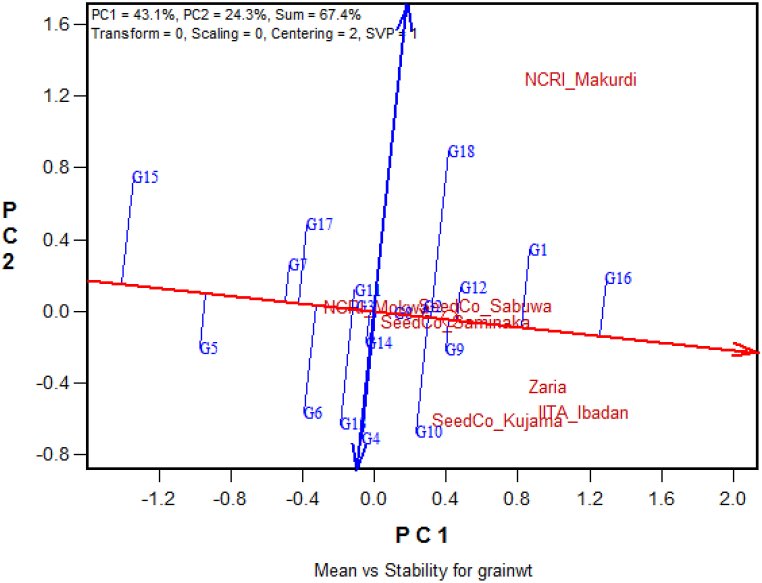
A biplot showing the mean performance and stability of the 15 soybean genotypes and three checks across seven test environments in Nigeria in 2019. PC: Principal Component.

**Fig. 4:**
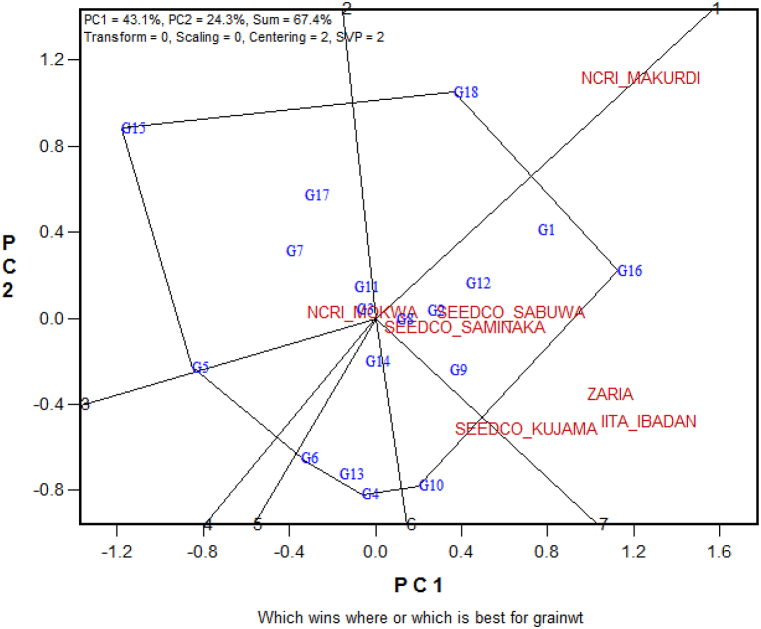
The which-won-where biplot of PC1 vs PC2 showing which of the 15 elite soybean genotypes and three checks performed best in which of the seven test environments PC = Principal Component.

The biplot that displays the mean yield performance and stability of the 18 soybean genotypes across seven test environments in Nigeria is presented in Fig. 3. Following the average-environment-coordinate (AEC) abscissa (the single-arrowed line), G16 has the highest performance for yield, followed by G1, G2, G9, G18, G10, and G12; while G4 and G14 produced the same mean grain yields with the grand mean. G15 was the lowest performing genotype followed by G5. The AEC ordinate (the double-arrowed line) displays the stability of the genotypes, and accordingly, G8, G2, G9 and G12 were some of the highly stable genotypes with above-average mean performances, whereas G18, G15, and G10 were highly unstable (Fig. 3). The highest-yielding genotype, G16, was also a stable genotype with a low projection distance from the AEC abscissa line, compared to G1, the highest-yielding check variety. G16 produced the highest mean grain yield performance (Table 3 and Fig. 3) and was the winning genotype in the cluster that contained several locations (Fig. 4), displaying good stability with a small projection distance from the AEC abscissa.

### 3.4 ‘Which-won-where’ polygon view of GGE biplot

The “which-won-where” biplot that displays the genotypes that performed best in which environments is presented in Fig. 4. A polygon was constructed by connecting the genotypes G18, G16, G10, G4, G5 and G15 that were farthest from the origin of the biplot so that all other genotypes were accommodated within the polygon. The genotypes at the vertices of the polygon were considered the winning genotypes for the sector to which the vertex genotype belonged to. The vertex genotypes were also considered responsive as they possess the longest vector in their respective directions. In this study, seven perpendicular lines were drawn from the origin of the biplot to each side of the polygon dividing the polygon into seven sectors that also categorized the study environments into three mega-environments. G16 was the vertex genotype for the sector in which Sabuwa, Saminaka, Zaria, Kujama and Ibadan locations were situated, whereas G18 was the winning genotype for the sector into which Makurdi location belonged (Fig. 4). G15 was the winning genotype in the mega environment where only the Mokwa location was situated. The perpendicular lines represent the equality lines between adjacent genotypes that permit their visual comparison.

### 3.5 AMMI analysis for variance components, overall performance and predicted means

In this study, the AMMI model analysis was utilized to offer a comprehensive and insightful overview of genotype-by-environment interactions (GEIs), while also exploring the relationships and variations in genotype performance under several environmental conditions. The AMMI analysis indicated high significant genotype, environment and genotype by environment effects in the soybean trials across the seven environments (Supplementary Table S1). The individual analysis revealed that five of the seven environments (∼71 %) showed significant differences in the genotype effect. The block effect was significant for only three (∼22 %) environments (Supplemental Table S2). The estimates of broad sense heritability ranged from 1.02 % for Mokwa to 87.40 % for Zaria (Supplemental Table S2). The genotypic accuracy of selection (As), which measures the correlation between predicted and observed values ranged from 0.10 for Mokwa to 0.93 for Zaria (Supplemental Table S2).

### 3.6 AMMI biplot interpretation

**Fig. 5:**
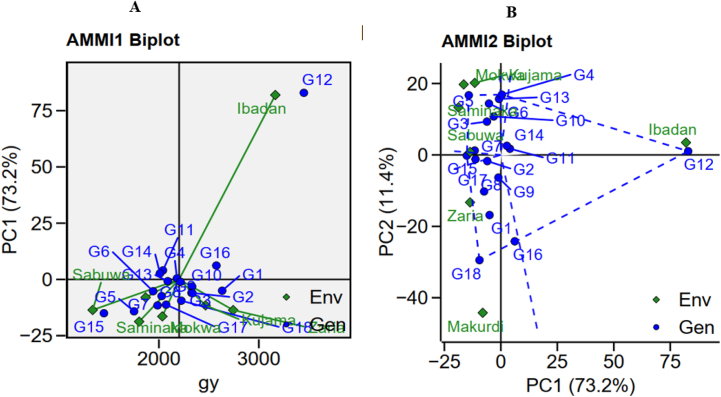
AMMI Biplot for grain yield using means of 15 soybean genotypes in seven environment against respective PC1 (A); AMMI Biplot for grain yield using their respective PC1 and PC2.

Ibadan exhibited the highest soybean GY mean (3165.5 kg ha^−1^), followed by Zaria (2743.5 kg ha^−1^) and the least was Sabuwa (1337.4 kg ha^−1^) (Fig. 5A; Supplementary Table S3). The first two IPCA accounted for 84.6 % of the total GEI variation (Fig. 5B; Supplementary Table S1). Among the seven environments evaluated, four (Sabuwa, Saminaka, Mokwa and Kujama) had a positive correlation, since the angle among them was <90°. This suggests that the magnitude of the interaction effects tended to be the same independently on the genotypes (Fig. 5B). Negative correlations indicated by vector angles >90° were observed in the locations Zaria, Markudi and Ibadan (Fig. 5B).

### 3.7 Genotype ranking for stability and performance

**Fig. 6:**
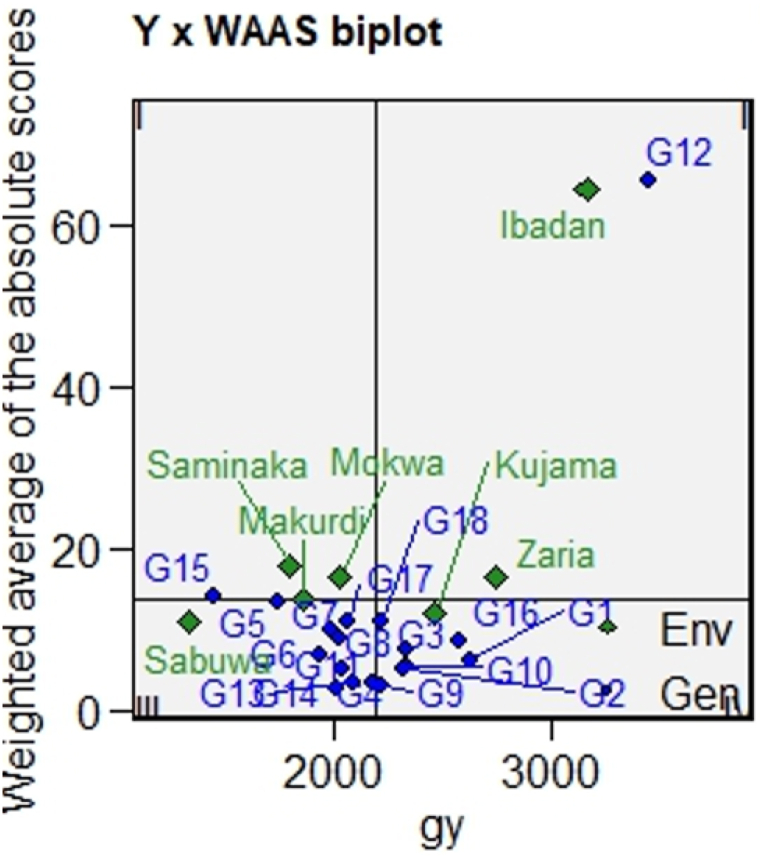
Biplot of 15 soybean genotypes evaluated in seven environments.

The quadrants in Fig. 6 represent the four classes of genotypes’ environment for a joint interpretation of performance and stability. In the first quadrant, the most unstable genotypes, the ones that contribute much to GEI and environments with high discrimination ability are included. The magnitude of the response variable (i.e., GY), however, is below the grand mean. The soybean genotype, G15, was in this quadrant. The GY of this genotype is far from the grand mean, and it presented the highest WAASY values (Supplementary Table S3). In the second quadrant, highly productive, but unstable genotype G12 is included. The environments included in this quadrant deserve attention since, in addition to providing high magnitudes of the response variable, they present a good discrimination ability of the genotype. For instance, in Ibadan, the mean GY of genotype G12 was higher than the grand mean of GY, indicating a higher discriminating ability of G12 (Fig. 6). The third quadrant consisted of low-productive and wide-adapted genotypes due to the lower values of WAASY (Supplementary Table S3). The lower this value, the more stable the performance of a genotype across environments. The environments included in this quadrant can be considered poorly productive and with low discriminating abilities. The genotypes within the fourth quadrant have above-mean productivity and lower values of WAASY (broadly adapted) (Supplementary Table S3). The environments included in this quadrant, however, can be considered productive but with low discrimination abilities. Among the genotypes in this quadrant are G9, G10 and G16 (Fig. 6).

**Fig. 7:**
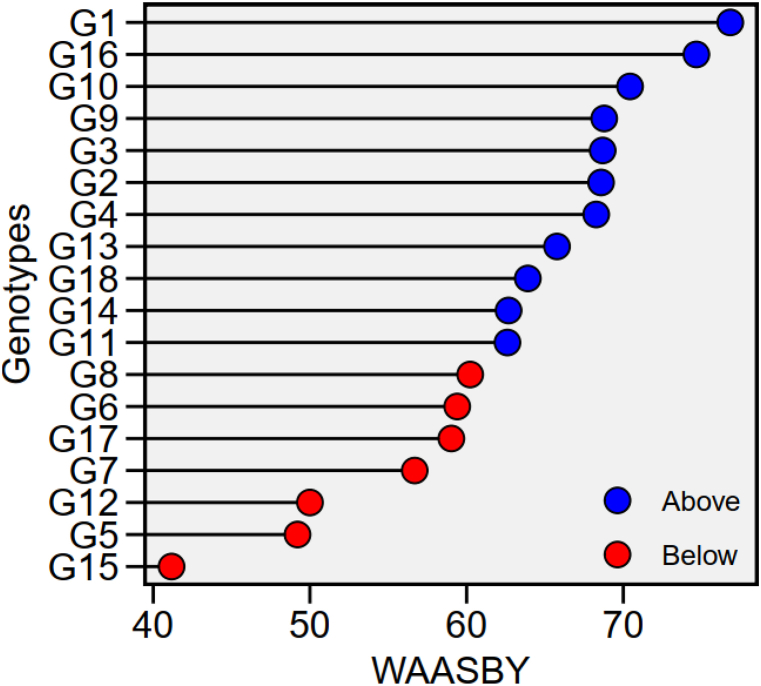
Estimated values of weighted average of the stability (WAASB) and mean performance (Y) (WAASBY) for 15 soybean genotypes.

**Fig. 8:**
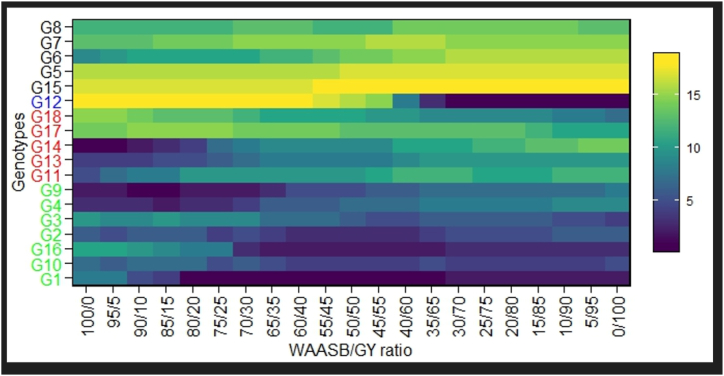
Ranks of 15 soybean genotypes considering different weights for stability and yielding.

The genotypes with higher WAASBY values were considered to be highly productive and stable (Fig. 7; Supplementary Table S3). The genotypes G16, G10, G9, G4 and G2 are in the category of genotypes with higher WAASBY values. The ranking of the genotypes based on WAASBY/GY ratio is presented in Fig. 8. The genotypes coloured in green were classified as the productive and broadly adapted genotypes (G16, G10, G9, G4 and G2) (Fig. 8). The genotypes coloured in blue were classified as the productive but not stable genotypes (G12). The genotypes with blue colour were classified as the stable, but unproductive genotypes (G11, G13, G14, G17 and G18). The genotypes coloured in black were classified as neither stable nor productive genotypes (G6, G7, G8, G17 and G18) (Fig. 8). Similarly, Zaria, Kajuma, Mokwa, Markudi and Saminaka are stable environments, which are less discriminating. In addition, the highest yield was recorded in Ibadan (Supplementary Table S2). Based on AMMI stability value (ASV) with lower ASV were considered as the highly adapted and stable genotypes (G9, G13, G4, G14 and G10), while higher ASV (G12 and G15) were considered as the unproductive and unstable genotypes (Supplementary Table S3).

## 4 Discussion

### 4.1 GGE model analysis

The identification of superior crop varieties with high yield and stability in various environments is the primary target of any crop improvement programs. This study highlights the significant differences among locations, genotypes and their interactions that signify the need to further assess the suitability of the studied soybean genotypes for either broad or specific adaptation. The response of soybean genotypes is greatly influenced by the specific location, as well as variations in annual temperature and rainfall [27,36]. Previous studies have also examined the impact of genotype, environment, and genotype × environment interaction (GEI) and reported significant differences among soybean genotypes and genotype × environment interaction for grain yield [27,[36], [37], [38], [39]]. Further, GEI poses challenges to plant breeders in selecting superior genotypes for adaptation to wider and diverse environmental conditions and commercial farming [9]. In this study, a higher proportion of the total variation was explained by GEI, compared to the genotypic variation (Table 6). The results corroborate with the results of Hilemariam [27], and Mwiinga [36] who had earlier reported that a higher proportion of the total variation was explained by GEI relative to the genotypic variation, which, according to Yan and Rajcan [40], and Yan and Kang [13], might indicate the possibility of identifying mega environments among the test environments. The environmental (locations) variation imposed a huge influence on the performance of the soybean genotypes for yield and accounted for 31.6 % of the total variation, while the GEI contribution (30.1 %) was nearly the same with the variation due to environment, suggesting that both sources of variation played greater roles in the performance of the soybean genotypes in this study (Table 6). In a similar study conducted in the Southern Africa region on the IITA soybean genotypes, Mwiinga [36] reported a higher (47.4 %) contribution of GEI, while the genotypic and environmental variations contributed 31.6 and 21.0 % to the total variation, respectively. Wijaya et al. [41] reported a huge contribution of genotype effect on the total GEI variation in a study conducted on grain yield stability study conducted on black soybean lines across three agroecosystems in West Java, Indonesia. In partitioning the environmental variances, the predictable (locations) component was a very important and significant source of variation [9]. Plant breeders can select genotypes for a given environment or substantially change genotypes across several environments when GEI is subjected to the action of predictable components [42].

In this study, the first two principal components of the GGE biplot explained 67.4 % of the variation, slightly surpassing the ideal limit of 66 % [20,36] of the total variation explained by the first two IPCAs in a study with a different set of soybean genotypes. However, our result was lower than that of Hailemariam [27], Arega et al. [43], Amira et al. [44], Carvalho et al. [45], and Bhartiya et al. [46] who reported 74.3, 93.6, 86.6, 80.0 and 74.4 %, respectively, but higher than the findings of Kocaturk et al. [47], Krisnawati and Adie [48], and Mulugeta et al. [49], which are 51.9, 60.9, and 63.4 %, respectively. The GGE biplot is crucial for assessing the discriminating ability and representativeness of the testing environments. It provides unbiased and valuable information about the tested genotypes [50,51]. In this study, Zaria and Ibadan were identified as the most discriminating and representative environments compared to Makurdi and Kujama. Their long vectors and acute angles with AEA contribute to their suitability in selecting broadly adapted soybean genotypes. Hashim et al. [52] reported that only environments with high discriminating abilities and representativeness are suitable for selecting genotypes for yield performance. Additionally, Yan and Tinker [32] and Arega et al. [53] reported that the length of the environmental vector is directly related to the standard deviation within the respective environments. Therefore, the length of the vector can be used to determine the discriminating ability of an environment, where a longer vector indicates a higher discriminating ability.

Two criteria i.e., the presence of different winning genotypes in different sectors [54], and the presence of significantly higher between-group variation than within-group variation [42], can be used to identify the mega environments. The polygon is useful in making comparison among the vertex genotypes [50]. The genotypes located at the vertices of the polygon of the ‘which-won-where’ biplot are considered the most responsive genotypes, as they are the furthest away from the origin in their respective directions [32]. In this study, three mega-environments were identified and the top-yielding genotype G16 was the winning genotype in the mega-environment that contained three locations: Sabuwa, Saminaka, Zaria, Ibadan, and Kujama (Fig. 4). Except Ibadan, all these locations were situated in the same agro-ecological zones (Fig. 1). The other mega environment, where G18 was the winning genotype, contained only one location i.e., Makurdi, which was situated in isolation on the agro-ecological map (Fig. 1). No environment was located in the sectors where G10, G4 and G6 were the vertex genotypes, indicating these genotypes performed poorly in any of the environments, which also indicates these genotypes were the poorest in some or all of the environments. However, according to Yan et al. [51], it is not enough to simply group environments; there must also be a repeatable pattern of "which-won-where" to designate mega-environments. This pattern ensures that genotypes such as G4 and G6, which performed poorly in all the environments, were properly identified [55].

All the yield performance indicators in this study revealed that G16 (TGx1989-19FxTGx1987-10F-5-3-2-2-3-I) showed higher performance than the IITA best check variety, G1. However, the yield of G16 was not significantly different from the IITA check variety, G1 (TGx 1951-3F). This is understandable as the top two checks, G1 (TGx 1951-3F) and G8 (TGx 1989–19F) were among the most prolific soybean varieties released and registered in Nigeria in the year 2014, with yield potentials of 2.5 and 3.0 t ha^−1^, respectively (Nigerian Seed Portal, https://www.seedportal.org.ng, verified June 1, 2020). The top yielder genotype (G16) also produced the highest individual location performance of 3566.6 kg ha^−1^ at Ibadan. G16 was also the winning genotype in the mega environment where the highest number of locations (three) were situated. In addition, G16, with its relatively smaller projection from the AEA abscissa axis showed a relatively more stable performance compared to the IITA best check variety, TGx 1951-3F. Due to the good performance of G16 for most of the desired parameters, such as high yield performance, good stability for diverse environmental conditions, large seed size, good nitrogen-fixing capacity, and tolerance to shattering, it can be considered a potential candidate variety for release and registration for enhanced soybean production in Nigeria.

### 4.2 AMMI model analysis

Application of the AMMI model for analyzing genotype by environment interaction (GEI) in a study involving 15 soybean genotypes across seven environments demonstrated that the first three IPCA of AMMI were statistically significant (Supplementary Table S1). This suggests that the interaction between genotypes and environments was effectively captured by the first three principal components. These results align with previous findings by Hailemariam [27] and Goa et al. [56]. However, they differ from the conclusions drawn by Zobel et al. [57] and Abiriga et al. [58], who suggested that accurate predictions could be achieved using only the first two IPCAs. In this study, GEI accounted for the largest proportion of treatment sum of squares (15.8 %), followed by environments (7.8 %) and genotypes (3.8 %), underscoring the importance of conducting multi-environment trials. Some studies have emphasized the significant contribution of GEI to overall variation [37,59], while others have highlighted the environment as the predominant source of variation [60,61].

The study utilized AMMI stability values (ASV) and WAASY scores to categorize genotypes based on their stability. Genotypes G10, G9, and G4 were identified as highly adapted and stable, while genotypes G5, G12, and G15 were determined to be highly unstable. Notably, the genotypes with the highest and lowest yields (G15 and G12) were both classified as unstable based on the stability indices. The AMMI1 biplot and stability measures like ASV and WAASY have proven effective in assessing stability in various research settings [62]. These results suggest that these stability parameters are valuable for selecting genotypes that exhibit both high yield and stability simultaneously [34].

## 5 Conclusion

The aim of this study was to evaluate the stability and adaptability of eighteen soybean genotypes using GGE biplot and AMMI biplot analyses. Significant differences were found among genotypes, environments, and genotype × environment interaction (GEI) for grain yield. The GGE and AMMI analysis models showed the importance of developing adaptable, stable, and high-yielding soybean genotypes across multiple environments. The GGE biplot analysis identified two mega-environments, with Makurdi having the highest grain yield stability and performance for genotype G18 and locations Zaria, Ibadan, and Kujama showing the highest grain yield stability and performance for genotype G16. The genotypes G2, G4, G9 and G10 were found to be most stable and adapted genotypes across the study locations. These genotypes could be used as parent materials for hybridization and commercial production due to their high grain yield potential. The differences in grain yield can be attributed to the genetic variation of the genotypes for yield performance across different environments and their interaction with the different soybean testing environments in Nigeria.

## Funding

This study was funded by USAID.

## Data availability statement

Data was made available in the IITA public repository (https://data.iita.org/), and the DOI of the data is: https://doi.org/10.25502/s857-9h71/d.

## CRediT authorship contribution statement

**Abush T. Abebe:** Writing – review & editing, Writing – original draft, Visualization, Validation, Supervision, Software, Resources, Project administration, Methodology, Investigation, Funding acquisition, Formal analysis, Data curation, Conceptualization. **Adeyinka S. Adewumi:** Writing – review & editing, Writing – original draft, Visualization, Validation, Software, Methodology, Investigation, Formal analysis, Data curation. **Moses Adeolu Adebayo:** Writing – review & editing, Writing – original draft, Visualization, Validation, Supervision, Resources, Project administration, Methodology, Investigation. **Aondover Shaahu:** Writing – review & editing, Writing – original draft, Visualization, Validation, Supervision, Resources, Project administration, Methodology, Investigation. **Hapson Mushoriwa:** Writing – review & editing, Writing – original draft, Visualization, Validation, Supervision, Resources, Project administration, Methodology, Investigation. **Tunrayo Alabi:** Writing – review & editing, Writing – original draft, Visualization, Validation, Software, Project administration, Methodology, Investigation, Formal analysis. **John Derera:** Writing – review & editing, Writing – original draft, Visualization, Validation, Supervision, Resources, Project administration, Methodology, Investigation, Funding acquisition. **Afolabi Agbona:** Software, Methodology, Formal analysis, Data curation. **Godfree Chigeza:** Writing – review & editing, Writing – original draft, Visualization, Validation, Supervision, Resources, Project administration, Methodology, Investigation, Funding acquisition.

## Declaration of competing interest

The authors declare that they have no known competing financial interests or personal relationships that could have appeared to influence the work reported in this paper.
