# Supplementary material for: Genotype x environment interaction and yield stability of soybean (Glycine max l.) genotypes in multi-environment trials (METs) in Nigeria
Source: Heliyon. 2024 Sep 18;10(19):e38097. doi: 10.1016/j.heliyon.2024.e38097 (PMC11470596; doi:10.1016/j.heliyon.2024.e38097)
Supplement: Multimedia component 1 [file mmc1.docx]

Table S1. Estimated variance components using AMMI for grain yield of 15 soybean genotypes evaluated in seven environments

| Source | DF | Mean Square | F Value | Pr (>F) | % Contribution | Proportion | Cummulative |
| --- | --- | --- | --- | --- | --- | --- | --- |
| ENV | 6 | 21052177.6 | 10.296 | 1.16E-07 | 7.78% | NA | NA |
| Block(Env) | 56 | 2044656.4 | 1.356 | 6.70E-02 |  | NA | NA |
| GEN | 17 | 3633487.1 | 2.411 | 2.03E-03 | 3.81% | NA | NA |
| GEN:ENV | 102 | 2512792.4 | 1.667 | 1.89E-03 | 15.79% | NA | NA |
| PC1 | 22 | 25581799.1 | 16.97 | 0.00E+00 |  | 73.2 | 73.2 |
| PC2 | 20 | 4373194.3 | 2.9 | 1.00E-04 |  | 11.4 | 84.6 |
| PC3 | 18 | 3869266.1 | 2.57 | 8.00E-04 |  | 9.1 | 93.6 |
| PC4 | 16 | 1707542.8 | 1.13 | 3.30E-01 |  | 3.6 | 97.2 |
| PC5 | 14 | 951369.3 | 0.63 | 8.38E-01 |  | 1.7 | 98.9 |
| PC6 | 12 | 697029.2 | 0.46 | 9.36E-01 |  | 1.1 | 100 |
| Residual | 196 | 1507353 | NA | NA |  | NA | NA |
| Total | 479 | 3388817.5 | NA | NA |  | NA | NA |

DF=Degree of Freedom, Env= Environment, Gen=Genotype, Pr (>F) = Probability of (greater than F value)

Table S2. Grand mean, mean squares for genotype, block and error of soybean genotypes across seven environments based on AMMI

| ENV | MEAN | MSG | MSB | MSE | AS |
| --- | --- | --- | --- | --- | --- |
| Ibadan | 3165.46 | 13469911.33ns | 11063926.55ns | 9227829.77 | 0.56 |
| Kujama | 2467.32 | 685140.72*** | 410397.06ns | 192771.47 | 0.85 |
| Makurdi | 1867.13 | 1912364.52*** | 1461074.94*** | 264945.85 | 0.93 |
| Mokwa | 2034.72 | 424025.74ns | 202469.62ns | 419681.30 | 0.10 |
| Sabuwa | 1337.36 | 190616.06ns | 704036.56*** | 133020.38 | 0.55 |
| Saminaka | 1802.60 | 470841.06*** | 268684.56* | 116612.85 | 0.87 |
| Zaria | 2743.51 | 1557341.80*** | 202005.35ns | 196609.18 | 0.93 |

Env= Environment, MSG=Mean square of genotype, MSB=Mean square of block, MSE=Mean square of error, AS=Genotype Accuracy of Selection

Table S3. Mean grain yield, IPCA score, WAASY, and ASV for 15 Soybean genotypes evaluated across seven environments.

| Genotype | Yield | PC1 | PC2 | PC3 | PC4 | PC5 | PC6 | WAASY | RWAASY | ASV | ASV_R |
| --- | --- | --- | --- | --- | --- | --- | --- | --- | --- | --- | --- |
| G1 | 2633.4 | -5.0 | -16.8 | -2.5 | -3.6 | 12.6 | 5.0 | 76.6 | 1 | 36.3 | 7 |
| G10 | 2332.3 | -3.1 | 10.8 | -18.3 | -2.5 | 4.6 | -3.7 | 69.6 | 4 | 22.9 | 5 |
| G11 | 2040.5 | 4.0 | 1.7 | 17.7 | 5.6 | 3.6 | -19.8 | 62.7 | 10 | 25.9 | 6 |
| G12 | 3450.9 | 82.9 | 1.0 | 5.0 | -7.0 | 0.3 | 3.7 | 50.0 | 16 | 533.6 | 18 |
| G13 | 2088.9 | -0.8 | 15.7 | -7.8 | 3.1 | -9.3 | -2.1 | 65.3 | 8 | 16.5 | 2 |
| G14 | 2011.0 | 2.7 | 2.5 | 0.8 | 3.6 | 0.4 | 1.0 | 64.0 | 9 | 17.4 | 4 |
| G15 | 1450.2 | -15.0 | -0.2 | 22.8 | -13.4 | -12.5 | 6.2 | 40.9 | 18 | 96.8 | 17 |
| G16 | 2575.1 | 6.2 | -24.2 | -6.7 | 19.2 | 0.4 | -0.9 | 73.4 | 2 | 46.4 | 11 |
| G17 | 2072.1 | -11.2 | -1.2 | 19.3 | 8.4 | -6.9 | 2.5 | 58.9 | 13 | 72.2 | 14 |
| G18 | 2224.5 | -9.5 | -29.5 | 0.9 | -13.1 | -4.9 | -6.9 | 62.5 | 11 | 67.6 | 13 |
| G2 | 2327.2 | -6.0 | -1.7 | 3.5 | 22.5 | 5.5 | 10.6 | 69.7 | 3 | 38.5 | 9 |
| G3 | 2335.3 | -6.1 | 9.3 | 14.3 | -1.1 | 16.1 | -2.4 | 68.3 | 6 | 40.6 | 10 |
| G4 | 2181.2 | 0.5 | 16.9 | -8.3 | 4.1 | -9.9 | 2.3 | 67.7 | 7 | 17.1 | 3 |
| G5 | 1751.4 | -14.2 | 16.7 | -0.6 | -10.1 | 11.4 | -4.7 | 49.0 | 17 | 92.8 | 16 |
| G6 | 1942.3 | -5.3 | 14.4 | -11.3 | 2.4 | -1.7 | 2.2 | 58.8 | 14 | 36.7 | 8 |
| G7 | 1986.6 | -11.6 | 1.2 | 6.6 | -7.1 | -3.1 | 11.2 | 57.6 | 15 | 74.4 | 15 |
| G8 | 2027.6 | -7.4 | -10.2 | -20.1 | -12.2 | 5.6 | 6.2 | 59.3 | 12 | 48.7 | 12 |
| G9 | 2216.1 | -1.1 | -6.3 | -15.5 | 1.2 | -12.2 | -10.3 | 68.6 | 5 | 9.4 | 1 |

IPCA=Interaction Principal Component Axis, PC=Principal components, WAASY = superiority index, RWAASY=Ranking based on superiority index, ASV=AMMI stability value, ASV-R=Ranking based on AMMI Stability Value
